# Development of a Quantum Dot-Based Immunochromatographic Assay for Rapid and Sensitive Detection of Non-Medical Etomidate Abuse

**DOI:** 10.3390/bios16080404

**Published:** 2026-07-24

**Authors:** Xin Yan, Liwei Jiang, Xiaolong Zhang, Yizhe Zhao, Zixin Wan, Jun Ma, Chunhui Song, Yikai Wang, Xingliang Liu

**Affiliations:** 1 Beijing Key Laboratory of Psychoactive Substances Detection and Control, Beijing Narcotics Control Technology Center, Beijing 100164, China; yanxinbucm@126.com (X.Y.); 18610256315@163.com (L.J.); zhaoyizhezhe@163.com (Y.Z.); wan18810715139@163.com (Z.W.); majun_0817@163.com (J.M.); songchunhui2023@163.com (C.S.); 2 National Narcotics Laboratory Beijing Regional Center, Beijing 100164, China; 3 Science and Technology Research Center of China Customs, Beijing 100026, China; zhangxiaolongzj@sina.com.cn

**Keywords:** etomidate, rapid detection, fluorescent quantum dot microspheres, immunochromatographic test strip

## Abstract

Etomidate is an ultrashort-acting imidazole-derived intravenous anesthetic. The non-medical abuse of etomidate as a new psychoactive substance has emerged as a critical public health challenge globally, particularly among adolescents, necessitating sensitive, rapid detection methods for forensic analysis. This study developed a quantum dot-based immunochromatographic test strip and a corresponding rapid detection method for the sensitive detection of etomidate acid in urine. CdSe/ZnS fluorescent quantum dot microspheres were used as labeling probes combined with specific monoclonal antibodies, and the detection protocol utilized a portable fluorescence reader. The method’s validation demonstrated a linear range of 10–2000 ng/mL (y = −0.231lnx + 0.5241, r = −0.991), with intra-batch and inter-batch coefficients of variation below 12%. The spike recovery rates ranged from 88.24% to 95.69%. Cross-reactivity testing against 12 common drugs and structural analogs showed no interference. The detection limit of 10 ng/mL represents a substantial improvement over conventional colloidal gold strips and approaches LC-MS/MS sensitivity. Assays of real urine specimens confirmed that the immunochromatographic test strip and detection method possess favorable accuracy and reliability. This fluorescent quantum dot-based immunochromatographic assay provides rapid, quantitative, and highly specific etomidate screening suitable for on-site forensic applications, effectively bridging front-end rapid testing with confirmatory laboratory analysis.

## 1. Introduction

Etomidate (ETO), an ultrashort-acting imidazole-derived intravenous anesthetic, was first discovered by German scientists A. Doenicke et al. in 1976 [1]. It is valued in clinical practice for its rapid onset, short duration of action, and favorable hemodynamic stability during anesthesia induction and procedural sedation [2]. In recent years, as the global crackdown on traditional illicit drugs has further intensified, new psychoactive substances (NPSs) and a host of emerging drug substitutes have cropped up one after another. The abuse of ETO as an alternative drug has been documented globally, primarily via inhalation from e-cigarette cartridges or powdered drug mixtures containing the substance [3,4].

Preclinical studies have provided compelling evidence of ETO’s abuse liability. Conditioned place preference (CPP) tests in mice demonstrated rewarding effects at a minimum dose of 3 mg/kg, equivalent to the CPP-inducing dose of ketamine. Intravenous self-administration experiments in rats also confirmed stable reinforcing effects [5]. These results revealed the addictive properties and abuse risk of ETO. Chronic or excessive use induces irreversible organ damage: neurotoxicity manifests as neuronal apoptosis, cognitive impairment, memory deficits, and neurotransmitter dysregulation [6], while gastrointestinal homeostasis is disrupted [7]. Adrenocortical suppression—a well-documented adverse effect of ETO—occurs through inhibition of 11β-hydroxylase, leading to reduced cortisol and aldosterone production, and has been linked to increased mortality in critically ill patients [8]. Additionally, ETO abuse is associated with psychological disturbances, including anxiety, irritability, hallucinations, and violent behavior, as well as fatal outcomes from respiratory depression or overdose [9].

Globally, ETO abuse has evolved into a pressing public health challenge, particularly in regions with stringent regulation of traditional illicit drugs. This abuse epidemic has spread to adolescents and young adults, with incidents reported in schools and public spaces [4]. ETO abuse is associated with severe health and societal harms. Acute effects include dizziness, unsteady gait, tremors, myoclonus, and confusion, often leading to falls, injuries, and public safety incidents such as traffic accidents [3]. As an NPS with addictive potential and a tendency for abuse, it has been classified as a controlled drug in several countries. Given these new changes in the drug situation and the practical needs of anti-drug operations, relevant detection methods—especially rapid detection technologies—must be quickly enriched to support forensic analysis to effectively curb the spread of related drugs in a timely manner.

Immunochromatographic rapid detection methods based on lateral flow immunoassay technology are among the most commonly used rapid testing techniques due to their advantages of fast detection, simple operation, high specificity, and minimal sample consumption [10]. The test strips employed in this method demonstrate excellent anti-interference capability for different matrices, making them particularly suitable for rapid drug detection in complex matrices. Immunochromatographic rapid test strips for various drugs, including morphine, methamphetamine, and cannabis, have been successfully developed and applied in anti-drug enforcement operations, becoming important tools for public security agencies in drug case investigations [11,12,13]. Quantum dot is an emerging class of fluorescent nanomaterials, essentially fluorescent semiconductor nanocrystals [14]. Due to their unique physicochemical and optical properties, the use of quantum dots as labels in immunochromatographic assays offers numerous advantages over traditional labels such as colloidal gold, including high detection sensitivity, low requirements for excitation light sources, strong photostability, a wide quantitative range, and ease of popularization [15]. Accordingly, they are suitable for widespread application in the field of rapid drug detection.

Etomidate acid (ETA) is the major metabolite of ETO. Following biotransformation, ETO is mainly present as ETA in urine. In response to the severe abuse epidemic and public security requirements, this study aimed to develop a dedicated immunochromatographic test strip and establish a rapid detection method for ETA in urine, using quantum dots as labeling materials in combination with a portable handheld reader. It is expected to provide technical support for the handling of ETO’s misuse as a recreational drug.

## 2. Materials and Methods

### 2.1. Materials

CdSe/ZnS fluorescent quantum dot microspheres (QDMs) were purchased from Mifu New Materials Technology Co., Ltd. (Shenzhen, China). An ETO monoclonal antibody and ETA coating antigen were obtained from Tansheng Technology Co., Ltd. (Chongqing, China). ETA and other compound standards were purchased from the Third Research Institute of the Ministry of Public Security. N-hydroxysuccinimide (NHS), 1-Ethyl-3-(3-dimethylaminopropyl) carbodiimide (EDC), Bovine Serum Albumin (BSA), absorbent pads, semi-rigid polyvinyl chloride (PVC) sheets, glass fiber membranes, nitrocellulose membranes (NC membranes), and plastic card shells were purchased from Nbgen Co., Ltd. (Beijing, China).

### 2.2. Characterization of QDMs

#### 2.2.1. High-Resolution Transmission Electron Microscopy

The microstructure and internal distribution of QDMs were characterized using a high-resolution transmission electron microscope (HRTEM, JEM-2100F, JEOL, Tokyo, Japan) operating at an accelerating voltage of 200 kV. Prior to observation, the QDM aqueous dispersion was diluted to 0.1 mg/mL to avoid particle aggregation. A drop of diluted suspension was uniformly deposited on a carbon-coated copper grid, followed by natural air drying at room temperature without any staining treatment. The overall morphology, structural integrity, monodispersity, and the encapsulation state of internal QDMs were visually observed and recorded.

#### 2.2.2. Zeta Potential Analysis and Particle Size Measurement by Dynamic Light Scattering (DLS)

The surface zeta potential (ζ) of QDMs was determined via dynamic light scattering (Zetasizer Pro, Malvern, UK). The QDM aqueous dispersion (0.1 mg/mL) was prepared under neutral pH conditions (pH = 7.0) and stabilized for 5 min before testing. The surface electrostatic properties and colloidal stability of the microspheres were evaluated according to the zeta potential values.

The hydrodynamic particle size of QDMs was measured using the same instrument. The QDMs were dispersed in ultrapure water to prepare a homogeneous dispersion with a concentration of 0.1 mg/mL. The dispersion was ultrasonically treated for 3 min to eliminate tiny aggregates. All DLS tests were performed at a constant temperature of 25 °C with a fixed scattering angle of 173°.

### 2.3. Preparation of QDM-Antibody Conjugates

A 100 μL QDM suspension was sonicated for 2 min, followed by the addition of 100 μL of 2-(N-Morpholino) ethanesulfonic acid (MES) buffer (25 mmol/L, pH 6.0). The mixture was centrifuged at 16,000 rpm for 10 min and resuspended in MES buffer. This washing procedure was repeated twice. EDC and NHS solutions were prepared. An 8 μL volume of EDC and 8 μL of NHS at 20 mg/mL in MES buffer were added sequentially and incubated at room temperature for 20 min. The obtained QDM solution was washed twice to remove residual EDC and NHS for subsequent use. The 0.02 mg monoclonal antibody was added to the activated QDMs and incubated for 2 h, followed by blocking solution (10% BSA) treatment. The supernatant was discarded, and the pellets were resuspended and washed twice to remove unbound antibodies. Finally, the pellets were resuspended in 200 μL of resuspension buffer to obtain the QDM-antibody-labeled conjugates. Fluorescence emission spectra of QDMs and QDM-antibody-labeled conjugates were measured using a Varioskan™ LUX Multimode Microplate Reader (Thermo Fisher Scientific, Waltham, MA, USA).

### 2.4. Optimization of Strip Preparation

Glass fiber membranes were employed as sample pads and conjugate pads. Three different treatment conditions of sample pads were compared to evaluate their effects on the chromatographic system: untreated, phosphate buffer treatment, and treatment with phosphate buffer supplemented with surfactants and macromolecular proteins. NC membranes were equilibrated for 1 h at 40% relative humidity before assembly.

The effect of the coating antigen concentration on the assay performance was also investigated. Four different concentrations of ETA-BSA (0.4, 0.8, 1.0, and 1.2 mg/mL) were dispensed onto the T line of the NC membrane. Two series of ETA-spiked urine samples (from one adult individual) at concentrations of 0 ng/mL and 20 ng/mL were prepared and tested under the aforementioned different T-line concentrations to determine the optimal coating concentration. Goat anti-mouse IgG was immobilized onto the control line at a concentration of 0.2 mg/mL.

### 2.5. Detection Principle and Protocol

The quantum dot immunofluorescence test strip adopts a competitive immunoassay mode. CdSe/ZnS QDs conjugated with anti-etomidate monoclonal antibodies act as fluorescent probes that cross-recognize urinary etomidic acid via their similar core structures. Etomidic acid from urine competes with test line coating antigen for antibody binding sites. Specific immune complex formation between etomidic acid and QD-labeled antibodies leads to evident fluorescence quenching of CdSe/ZnS quantum dots. Higher etomidic acid content corresponds to weaker test line fluorescence, and the quantitative negative correlation between fluorescence signal intensity and target concentration enables accurate detection of etomidic acid in urine.

**Figure 1:**
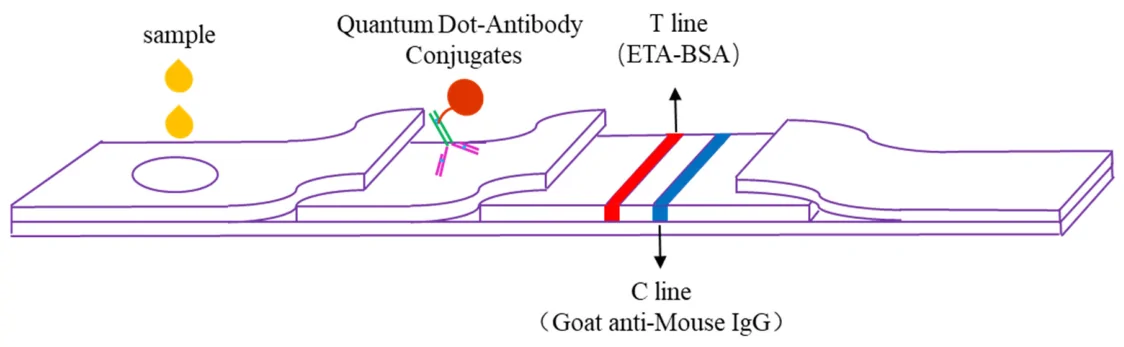
Schematic illustration of the QDM-based ETA immunochromatographic test strip and the urine sample analysis.

A 100 μL aliquot of the urine sample to be tested was applied to the sample port of the prepared test strip card. After a 10 min reaction, the test strip card was inserted into a fluorescence reader (FIC-H1, Nbgen Co., Ltd.). The fluorescence reader employs an excitation light source (365 nm) to stimulate fluorescent labels on immunochromatographic strips. The emitted fluorescence from T and C lines is collected and converted into digital signals. The result was determined based on the ratio of fluorescence intensity between the T line and C line (Figure 1).

### 2.6. Method Validation

#### 2.6.1. Evaluation of Linear Range

Blank urine from three different adults was individually spiked with ETA standard to prepare a series of standard solutions at concentrations of 10, 20, 50, 100, 200, 500, 1000, and 2000 ng/mL, serving as three biological replicates for detection. The standard curve was fitted using log(T/C) as the ordinate and the natural logarithm of the detected concentration as the abscissa to determine the linear range.

#### 2.6.2. Precision Assessment

Three batches of prepared test strips were randomly selected to evaluate precision. Blank natural urine samples from one adult individual spiked at different concentrations (50 and 500 ng/mL) were tested, with 10 replicates for each concentration per batch. Precision was assessed using the intra-batch and inter-batch coefficients of variation (CVs).

#### 2.6.3. Accuracy Assessment

ETA spiked natural urine sample (from one adult individual) solutions were prepared at a concentration of 20 ng/mL, to which 100 μL volumes of ETA solution at 200 ng/mL and 400 ng/mL were added, respectively. Additionally, ETA natural urine sample solutions at 200 ng/mL were prepared, to which 100 μL volumes of ETA solution at 2000 ng/mL and 4000 ng/mL were added, respectively. After spiking, the four sample solutions were tested in triplicate, and the accuracy was evaluated using recovery rates.

#### 2.6.4. Evaluation of Specificity

The cross-reactivity (CR) rate is a critical indicator reflecting the specificity of immunochromatographic rapid detection methods. The CR of twelve common drug components and structural analogs for the test strips was investigated according to previously reported methods [16].

### 2.7. Real Urine Specimen Analysis

Real urine samples were collected from 10 suspected etomidate abusers under routine drug screening at the National Narcotics Laboratory, Beijing Regional Center. All samples were residual routine testing specimens without additional intervention carried out on the individuals. The real urine samples were determined by the developed immunochromatographic test strip. Liquid chromatography−mass spectrometry (LC-MS/MS) was used to confirm urine specimens requiring verification. Quantitative analysis was performed on a Shimadzu LCMS-8060NX triple quadrupole mass spectrometer (Shimadzu, Kyoto, Japan). Data acquisition and processing were controlled by LabSolutions LCMS Workstation software. Chromatographic separation was achieved on a Shim-pack GIST C18 AQ column (2.1 mm × 50 mm, 1.9 μm, Shimadzu) maintained at 40 °C, with a mobile phase consisting of 0.1% formic acid in water (phase A) and acetonitrile (phase B) delivered at a flow rate of 0.4 mL/min. The gradient elution conditions were as follows: 5% B held for the first 1.0 min, linearly increased to 95% B from 1.0 to 8.0 min, maintained at 95% B until 9.0 min, rapidly returned to 5% B within 0.1 min, and re-equilibrated at 5% B for 1.9 min, resulting in a total run time of 11 min. Mass spectrometric detection was performed under electrospray ionization positive mode (ESI+) and multiple reaction monitoring (MRM) mode, with an interface voltage of 1.0 kV, an interface temperature of 350 °C, a desolvation line (DL) temperature of 250 °C, a nebulizing gas (N_2_) flow of 3.0 L/min, a drying gas (N_2_) flow of 10.0 L/min, and a heating gas (N_2_) flow of 10.0 L/min. The MRM transitions for ETA were m/z 217.1/95.0 (quantifier) and m/z 217.1/105.0 (qualifier), with collision energy (CE) optimized to 32 eV and 33 eV [17].

## 3. Results

### 3.1. Characterization Results of QDMs

#### 3.1.1. Morphology and Microstructure Analysis

**Figure 2:**
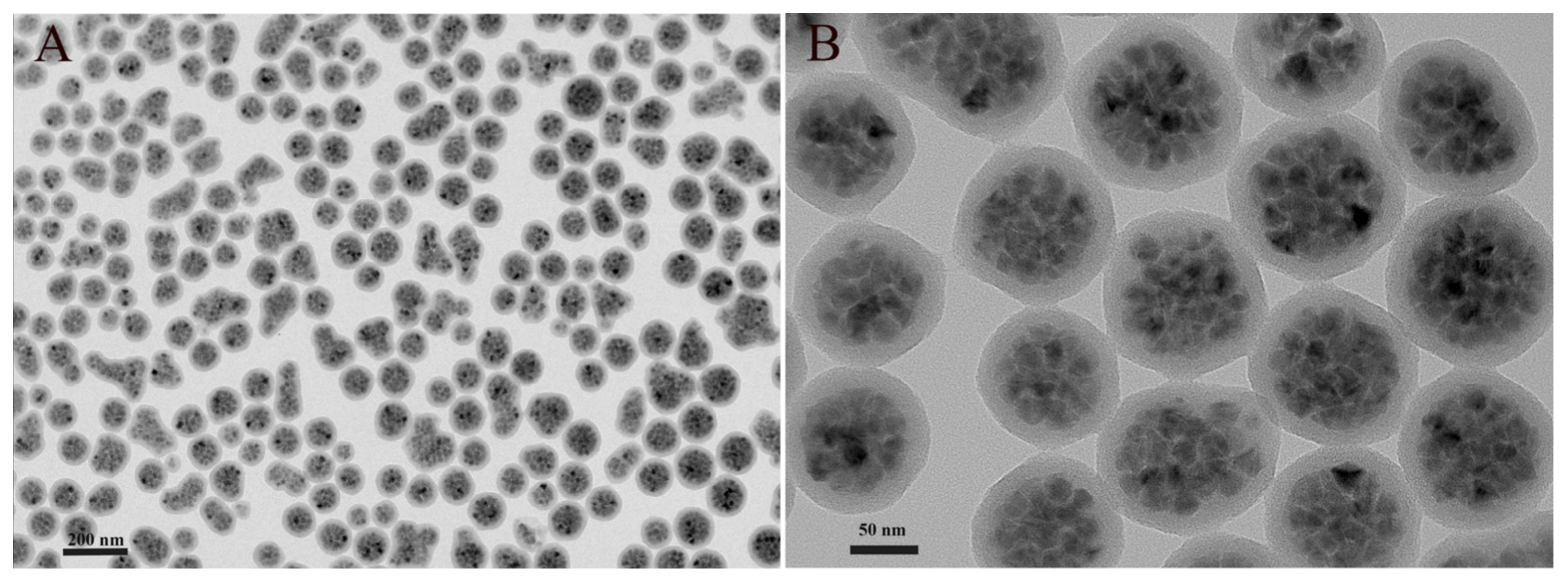
Morphology of QDMs dispersed in aqueous media: (**A**,**B**) HRTEM images of QDMs with different magnifications. The scale bars are 200 nm and 50 nm, respectively, for (**A**,**B**).

The HRTEM images intuitively demonstrated that the prepared QDMs presented a regular spherical structure with complete and smooth surface morphology, without obvious structural defects, collapse, or irregular deformation (Figure 2). Internally, numerous quantum dots were uniformly encapsulated inside the polymer microsphere matrix, with no obvious aggregation or uneven distribution of quantum dots observed. This uniform encapsulation structure effectively avoided the fluorescence quenching caused by quantum dot agglomeration and external environmental interference, which laid a solid foundation for the stable and high fluorescence signal output of the immunochromatographic assay. In addition, the microspheres exhibited good independent dispersion states, with no adhesion and cross-linking between adjacent particles, which was conducive to the uniform flow of probes on the nitrocellulose membrane in subsequent lateral flow detection.

#### 3.1.2. Particle Size and Zeta Potential

**Figure 3:**
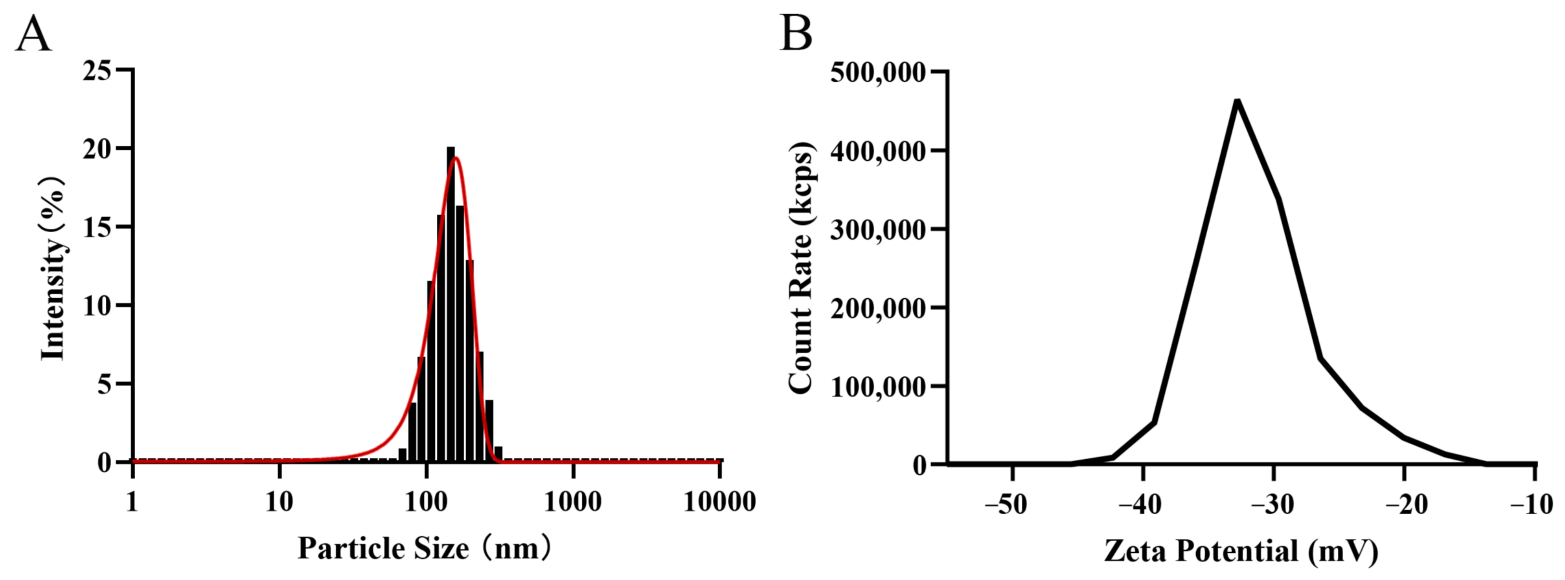
Dynamic light scattering (DLS) analysis of the QDMs: (**A**) hydrodynamic diameter distribution of the QDMs; (**B**) zeta potential distribution of the QDMs.

The DLS statistical results showed that the average hydrodynamic diameter of QDMs was 154.60 ± 48.51 nm (Figure 3A). The zeta potential of QDMs in neutral aqueous solution was measured to be −31.31 ± 4.41 mV (Figure 3B). It is generally accepted that a zeta potential absolute value greater than 30 mV indicates strong electrostatic repulsion between particles and excellent colloidal stability. The highly negative zeta potential of QDMs endowed the microspheres with good anti-aggregation ability in aqueous dispersion and a biological buffer environment. This stable colloidal property enabled the QDM-antibody conjugates to maintain uniform dispersion during the immuno-binding reaction and chromatographic migration process, preventing probe precipitation and non-specific adsorption on the test strip. Accordingly, the background fluorescence interference was reduced, and the sensitivity and signal stability of the non-medical etomidate abuse detection assay were significantly guaranteed.

#### 3.1.3. Fluorescence Emission Spectrum

**Figure 4:**
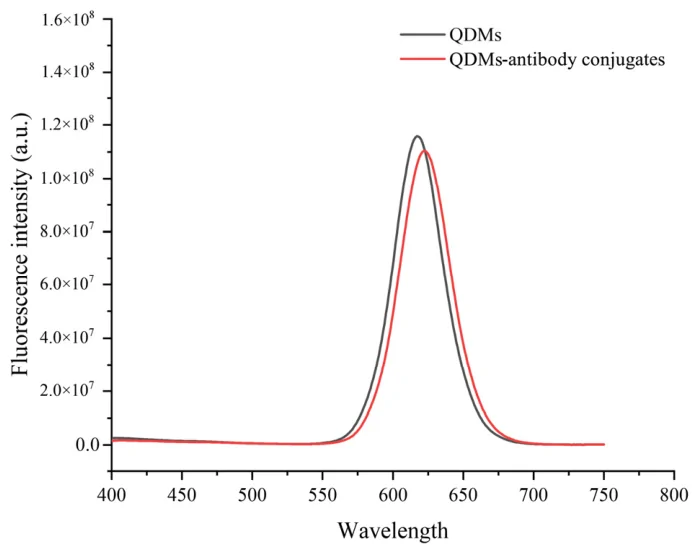
Fluorescence emission spectra of QDMs and QDM-antibody conjugates.

The QDM-antibody conjugates displayed an emission peak at 622 nm, in comparison with the maximum emission wavelength of 617 nm for pristine QDMs, indicating a negligible difference in emission wavelength (Figure 4).

### 3.2. Optimization of Test Strip Preparation Conditions

#### 3.2.1. Sample Pad Treatment

**Figure 5:**
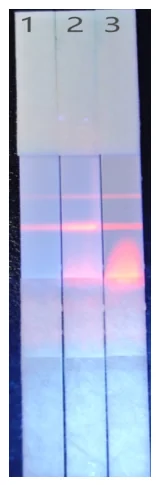
Chromatographic performance of test strips with different sample pad treatments.

As shown in Figure 5, Condition 3 (untreated) exhibited slow release with significant accumulation of microspheres; Condition 2 (treatment with 0.02 mol/L phosphate buffer) showed improvement but still displayed poor microsphere release and non-uniform chromatography on the NC membrane; Condition 1 (phosphate buffer supplemented with surfactants, macromolecular proteins, and preservatives) demonstrated optimal microsphere release and uniform strip formation.

#### 3.2.2. T Line Coating Conditions

Four different coating antigen concentrations were selected with a fixed coating volume to determine the optimal T line dispensing conditions. As shown in Table 1, when the coating concentration was 1.0 mg/mL, the T line exhibited the strongest fluorescence signal with an optimal gradient. A further increase in coating concentration did not result in additional gradient improvement.

### 3.3. Method Validation Results

#### 3.3.1. Linear Range

**Figure 6:**
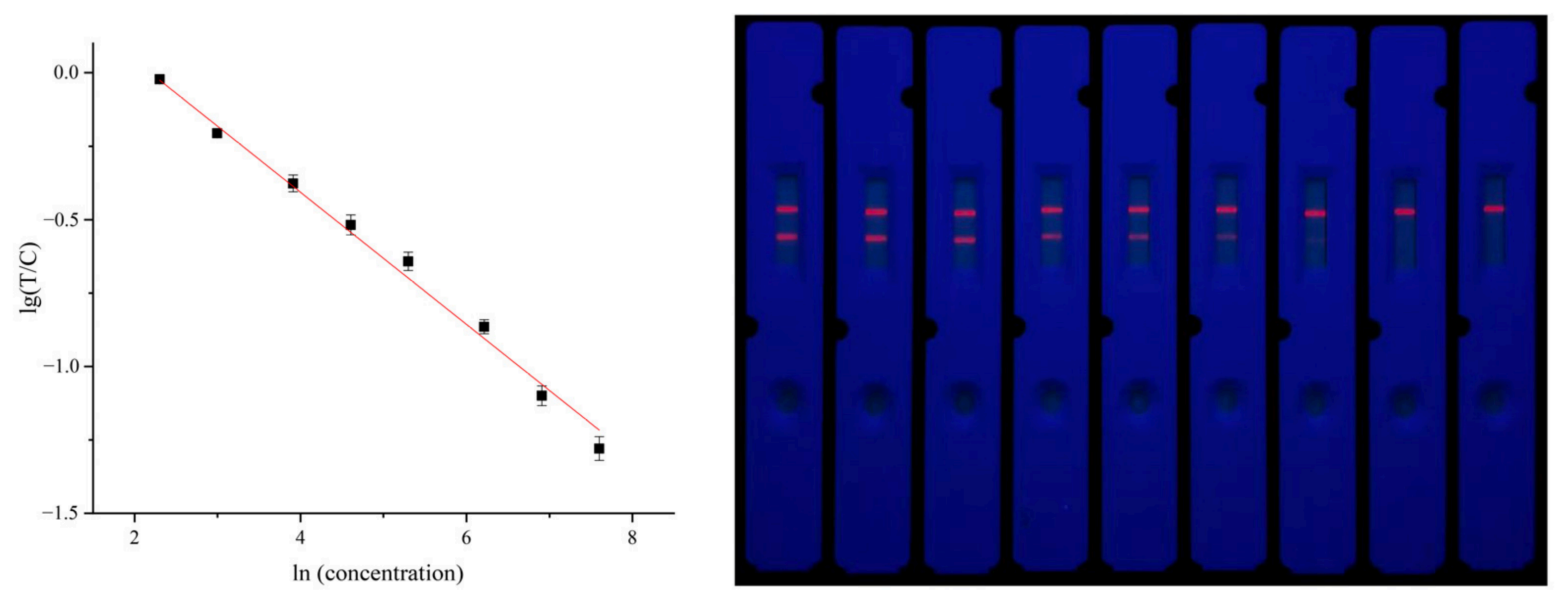
Establishment of ETO immunochromatographic test strip standard curve.

Serial concentration testing results showed that the T/C values decreased gradually with increasing ETA concentration. Using ln(concentration) as the abscissa and log(T/C) as the ordinate, the quantitative standard curve was established, as shown in Figure 6. Statistical fitting analysis yielded the equation y = −0.231lnx + 0.5241 (r = −0.991), demonstrating linearity in the range of 10–2000 ng/mL.

#### 3.3.2. Precision

Samples at two concentrations (50 ng/mL and 500 ng/mL) were tested using three different batches of immunochromatographic test strips to evaluate precision. As shown in Table 2, the intra-batch and inter-batch CV values for both concentration samples were less than 10% or 12%, indicating good precision.

#### 3.3.3. Spike Recovery

As shown in Table 3, four spiked urine sample solutions were tested using the test strips, with spike recovery rates of 95.46%, 94.24%, 88.26%, and 90.34%, respectively, all within the acceptable range of 85–115%. These results indicate good accuracy of the test strip and rapid detection method.

#### 3.3.4. Specificity

CR testing was performed to evaluate the specificity of the test strip and rapid detection method. Lower CR rates indicate higher strip specificity and more accurate method results. Twelve representative common drug components were selected for evaluation. The results showed no CR (Table 4), indicating no interference with detection.

### 3.4. Application in Real Urine Samples

Briefly, 10 real urine samples from suspected etomidate abusers were determined using both the developed immunochromatographic test strip and LC-MS/MS methods. As shown in Table 5, the results revealed a high consistency between the immunochromatographic test strips and LC-MS/MS analyses, with the relative accuracy ranging from 96.1% to 109.3%.

## 4. Discussion

The addictive properties of ETO, together with the severe health risks caused by its chronic abuse, underscore the importance of the screening and strict regulation of ETO abuse. China classified ETO as a Class II psychotropic substance in October 2023, implementing strict management protocols to curb diversion and abuse [18]. South Korea designated ETO under the “Regulation on the designation of drugs that may cause concerns of misuse or abuse” in June 2020, though this framework is less stringent than the country’s Narcotics Control Act [3]. In such control practices, rapid detection approaches featuring high sensitivity, speed, and convenience constitute indispensable technical support.

Colloidal gold represents the most classic and widely used label in immunochromatographic methods, with colloidal gold-based assays being the most common technical approach in current rapid drug detection products. However, due to their grayscale-based colorimetric discrimination mode, such rapid test strips have limitations in sensitivity, accuracy, and linear range, which cannot fully meet increasingly demanding requirements [19,20]. The continuous exploration of novel labeling materials represents the main development direction for this rapid detection technology. In recent years, numerous new labeling materials have been applied in immunochromatography, providing novel technical strategies for the advancement of immunochromatographic technology [10]. Fluorescent quantum dots, as novel labeling materials, exhibit superior optical performance compared to colloidal gold and organic fluorescent dyes, including a high quantum yield, strong resistance to photobleaching, a larger Stokes shift, and advantages in photoelectric signal mode compared to grayscale signals [15]. Due to these optical characteristics, quantum dot-based immunochromatographic methods offer many advantages over colloidal gold and other labeled probes, including higher detection sensitivity, a wider quantitative range, lower excitation source requirements, reduced background interference, and enhanced photostability, making them highly suitable for application in rapid drug detection.

Studies have demonstrated that ETO immunochromatographic rapid test strips developed based on conventional colloidal gold labeling technology can achieve a detection limit of 500 ng/mL. In contrast, the quantum dot labeling-based immunochromatographic rapid test strip developed in this study exhibits substantially improved sensitivity, with a detection limit far below that of the colloidal gold-based strips, further highlighting the advantages of quantum dot labeling technology in rapid drug detection applications. CR testing revealed no interference from common drug components with this test strip and rapid detection method, confirming its excellent specificity and the effective prevention of numerous false-positive results that could interfere with case judgment. Furthermore, because the quantitative limit of this method can reach 10 ng/mL, approaching the detection limit of LC-MS/MS methods [21], this test strip, as a front-end rapid screening technology, can better interface with back-end large instrument analytical techniques such as LC-MS/MS, providing high-matching guidance for the final identification and investigation of cases.

In view of the addictive potential and health hazards of etomidate and the urgent demand for regulatory control, relevant specific rapid detection techniques have attracted increasing research attention in recent years. Liu et al. fabricated a europium fluorescent microsphere-based lateral flow immunoassay (LFIA) with a broad-spectrum monoclonal antibody capable of simultaneous qualitative and quantitative analysis of etomidate and metomidate within 10 min in water, urine, and serum matrices [22]. The immunoassay possessed outstanding anti-interference capacity against various psychoactive compounds and showed far better sensitivity than conventional colloidal gold strips prepared with identical antibodies. As urine specimens from suspects mainly contain metabolized etomidate acid instead of the prototype drug, this assay may produce false-negative results for actual case samples, which greatly impairs its reliability in law enforcement screening. As an alternative non-immune fluorescent sensing route, Li et al. developed copper nanocluster molecular imprinted polymer (CuNCs@MIP) probes for etomidate detection [23]. The probe relied on specific imprinted cavities to capture etomidate and produced a fluorescence quenching response, with a linear range of 10–500 ng/mL in urine. In comparison, our rapid test strip constructed via a specific antibody-based immunoassay achieves better anti-interference specificity and an extended linear detection range.

## 5. Conclusions

This study successfully established a QDM-based immunochromatographic assay for rapid ETA detection in urine. The strip showed a wide linear range, favorable precision and accuracy, and high specificity without cross-interference from common drugs. It showed greatly improved sensitivity over conventional colloidal gold strips and enabled quantitative performance close to LC-MS/MS. Assays of authentic urine specimens confirmed that the QDM-based immunochromatographic test strip and the rapid detection method possess favorable accuracy and reliability. With simple operation and rapid detection, this method provides reliable technical support for on-site screening and forensic analysis of ETO abuse, effectively meeting the demand for the rapid monitoring of NPSs in anti-drug practice.

## Figures and Tables

**Table 1:** Optimization results for antigen coating concentration.

Coating Concentration (mg/mL)	T/C	
	0 ng/mL	20 ng/mL
0.4	0.582	0.414
	0.608	0.448
	0.593	0.437
0.8	0.791	0.603
	0.787	0.602
	0.780	0.597
1.0	0.958	0.642
	0.983	0.649
	0.976	0.645
1.2	1.002	0.679
	0.993	0.667
	1.018	0.676

**Table 2:** Precision evaluation results for the immunochromatographic test strips.

Sample Concentration (ng/mL)	CV (%)	
	Intra-Batch	Inter-Batch
50	9.03	11.92
500	5.80	8.28

**Table 3:** Urine spike recovery rates of the immunochromatographic test strips.

Sample	Spike Amount (ng)	Detected Amount (ng)	Recovery (%)
1	20	19.09	95.46
2	40	37.70	94.24
3	200	176.52	88.26
4	400	361.34	90.34

**Table 4:** CR test results of the immunochromatographic test strips.

Compound	IC_50_ (ng/mL)	Cross-Reactivity (%)
Methamphetamine	>10^5^	<0.01
Morphine	>10^5^	<0.01
Ketamine	>10^5^	<0.01
Methadone	>10^5^	<0.01
Tetrahydrocannabinol	>10^5^	<0.01
Cocaine	>10^5^	<0.01
Ephedrine	>10^5^	<0.01
Caffeine	>10^5^	<0.01
Mephedrone	>10^5^	<0.01
Fentanyl	>10^5^	<0.01
Diazepam	>10^5^	<0.01
Triazolam	>10^5^	<0.01

**Table 5:** Real urine specimen detection results of the immunochromatographic test strips and LC-MS/MS.

Sample	Test Strips (ng/mL)	LC-MS/MS (ng/mL)	Relativity (%)
1	ND	ND	100
2	149	155	96.1
3	ND	ND	100
4	ND	ND	100
5	1458	1408	103.6
6	176	161	109.3
7	272	260	104.6
8	ND	ND	100
9	700	720	97.2
10	400	385	103.9

Note: ND, not detected.

## Data Availability

The original contributions presented in this study are included in the article. Further inquiries can be directed to the corresponding authors.

## Abbreviations

The following abbreviations are used in this manuscript:

NPS	New psychoactive substances
QDMs	Quantum dot microspheres
ETO	Etomidate
ETA	Etomidate acid
NHS	N-hydroxysuccinimide
EDC	1-Ethyl-3-(3-dimethylaminopropyl) carbodiimide
MES	2-(N-Morpholino) ethanesulfonic acid
PVC	Absorbent pads, semi-rigid polyvinyl chloride
NC	Nitrocellulose
CVs	Coefficients of variation
BSA	Bovine Serum Albumin
IC_50_	Half-maximal inhibitory concentration
CR	Cross-reactivity
CPP	Conditioned place preference
